# Preoperative systemic inflammation and muscle fatty infiltration are prognostic factors for quadriceps atrophy following anterior cruciate ligament reconstruction

**DOI:** 10.3389/fimmu.2026.1796054

**Published:** 2026-03-25

**Authors:** Hanyi Wang, Yuqi Li, Hao Zheng, Beijie Qi, Jiwu Chen, Jian Xu, Hongbo Huang, Yaying Sun

**Affiliations:** 1 Department of Sports Medicine, Shanghai General Hospital, Shanghai Jiao Tong University School of Medicine, Shanghai, China; 2 Shanghai Jiao Tong University School of Medicine, Shanghai, China; 3 Department of Orthopedics, Shanghai Pudong Hospital, Fudan University Pudong Medical Center, Shanghai, China; 4 Shanghai General Hospital of Nanjing Medical University, Shanghai, China; 5 Department of Orthopaedics, Sports Medicine and Arthroscopy, Fuyang People’s Hospital Affiliated to Anhui Medical University, Anhui, China

**Keywords:** anterior cruciate ligament reconstruction, apoptosis, fibro/adipogenic progenitors, inflammation, meniscus repair, muscle atrophy, muscle fatty infiltration

## Abstract

**Background:**

Muscle atrophy following anterior cruciate ligament reconstruction (ACLR) significantly impedes functional recovery, yet its underlying prognostic factors and potential cellular mechanisms remain poorly understood.

**Methods:**

In a cohort of 26 ACLR patients, a correlation analysis was conducted between clinical characteristics and postoperative changes in quadriceps circumference. Single-cell RNA sequencing was performed to analyze cell composition and intercellular communication in immobilized muscle. Ligand–receptor interactions were investigated using CellChat, and ferroptosis level was evaluated in fibro/adipogenic progenitors (FAPs). *In vitro* experiments were conducted using apoptotic muscle satellite cells (MuSCs) conditioning medium to evaluate its effects on FAP activation and adipogenic differentiation.

**Results:**

Preoperative muscle fat infiltration (MFI), C-reactive protein (CRP) level, and concurrent meniscus repair were independent predictors of quadriceps atrophy at 3 months post-ACLR. Single-cell analysis revealed increased proportions of MuSCs and FAPs in immobilized muscle with enhanced cross-talk. Conditioned medium from apoptotic MuSCs promoted FAP viability and adipogenic ability. Moreover, ferroptosis was suppressed in FAPs under immobilization, with several regulatory genes aberrantly regulated. Mechanistically, immobilization induced a coordinated shift in the ferroptosis regulatory network within FAPs, characterized by downregulation of pro-ferroptotic genes and upregulation of anti-ferroptotic genes.

**Conclusion:**

Preoperative MFI and systemic inflammation are risk factors for quadriceps atrophy after ACLR. Apoptotic MuSCs may promote fat infiltration by activating FAPs through suppression of ferroptosis. These findings highlight potential therapeutic targets for mitigating muscle degeneration and improving recovery outcomes after ACLR.

## Introduction

Anterior cruciate ligament (ACL) rupture is a common sports-related injury. Anterior cruciate ligament reconstruction (ACLR) is a vital treatment for restoring knee stability and improving knee function in patients (1–3). To return to sports is the primary reason patients with ACL injuries opt for ACLR, especially those who participate in high-intensity or directional-change sports particularly may benefit from ACL reconstruction (2). Though surgical techniques have advanced, extensive clinical observations have indicated that patients still face challenges in achieving long-term functional recovery. Common contributing factors impacting knee function after ACLR include graft failure, knee fibrosis, and muscle atrophy (1, 4, 5).

Returning to sports after ACLR is a multifactorial outcome, depending on multiple factors. These factors include preoperative, intraoperative, and postoperative aspects (6). Restoring knee muscle strength after ACLR is essential for patients to return to sports (7, 8). Knee trauma or ACLR often leads to varying degrees of neuromuscular dysfunction in patients. Notably, neuromuscular activation deficits in the lower extremities persist for over three years post-ACLR in approximately 42% of cases (9). In the early postoperative period, subjective functional outcomes correlate more closely with poor recovery of quadriceps and hamstring muscle strength than with graft appearance or tension (5). Postoperative quadriceps atrophy and persistent quadriceps weakness are important factors limiting patients returning to sports (3, 9, 10). Preoperative quadriceps atrophy is common in ACL injuries (11), and this condition often persists after ACLR (12, 13).

Quadriceps dysfunction is prevalent after ACLR, and addressing this dysfunction is important for improving knee function and reducing the risk of re-injury (13, 14). Traditional views hold that skeletal muscle inevitably undergoes degenerative changes when exposed to pathological stimuli. These stimuli include aging and various injuries, leading to a decline in muscle dimensions and strength (15–19). The reduction in quadriceps strength after ACL injury used to be primarily attributed to disuse-induced muscle atrophy (20). However, recent advances in imaging technology have shifted this understanding. Researchers now have recognized that fat infiltration is one of the key contributor to the widespread muscle wasting observed after ACLR (21). It is well established that MFI impairs skeletal muscle contraction. It is also closely associated with numerous pathophysiological features, including skeletal muscle atrophy, fibrosis, and impaired energy metabolism (22, 23).

Increasing evidence indicates that fat content in skeletal muscle progressively increases during the degenerative process (22, 24). Fat infiltration involves chronic alterations in the muscle microenvironment. This condition exhibits poor reversibility and posing greater challenges for recovery (17, 25, 26). Worse still, fat infiltration may compromise the dynamic joint stability and alter load distribution, making it a potential risk factor for secondary knee osteoarthritis (27–29). Notably, preoperative MFI may be more than a baseline risk factor—it could reflect an ongoing pathological predisposition. Therefore, investigation into the patterns of muscle atrophy and fat infiltration development after ACLR holds significant clinical significance for overcoming current rehabilitation bottlenecks and improving long-term patient outcomes. In the current study, we organized a preliminary cohort of ACLR. By unveiling the link between pre- and post-operative measurements, the clinical significance of MFI on recovery was confirmed.

## Methods

### Participants

Data involving human subjects were approved by the Ethics Committee of local hospital (2022KY067) in accordance with the principles of the Declaration of Helsinki. Written informed consent was obtained from all cases. This study recruited patients who underwent primary unilateral ACLR at our local institution in the third quarter of 2024. Eligible participants met the following criteria: (1) aged 18 to 60 years, and (2) free from other knee conditions or surgeries besides the current ACL injury. Exclusion criteria included: (1) Concurrent participation in other drug or exercise trials, (2) Presence of other conditions limiting lower limb function and range of motion and/or interfering with functional assessment, (3) Any contraindications for MRI. Included participants underwent preoperative blood draws for laboratory blood counts to obtain C-reactive protein, neutrophil, and lymphocyte values. MRI scans of the thigh were performed within one week prior to surgery, and thigh circumference measurements were taken one day before surgery at the specific location 5 cm above the patella. All examinations were conducted after obtaining informed consent from the patients.

MRI Assessment of quadriceps muscle fatty infiltration.

Muscle MRI was performed on patients at 1.5 Tesla to assess thigh positioning following ACLR surgery. Images were acquired in the axial plane using T1-weighted, Short Tau Inversion Recovery (STIR), and Fast Spin Echo (FSE) sequences. Muscle fat infiltration was defined in T1-weighted MRI sequences. The ratio of fat infiltration within the quadriceps femoris muscle of ACLR patients was measured using an open-source algorithm reported by Tam et al. (30), which enables fully automated muscle segmentation and quantification of intramuscular fat infiltration levels from MRI images with high reproducibility and accuracy.

Acquisition and induction of MuSCs.

Skeletal muscle satellite cells were purchased from Procell Inc. (Wuhan, China) and maintained under standard culture conditions according to the protocol. Then, MuSCs were treated with 0.5 µM staurosporine (Beyotime, Shanghai, China) for 12 hours to induce apoptosis. The cells were cultured in serum-free DMEM for 24h to collect the supernatant. Larger cellular debris were removed by centrifugation at 800 g for 10 min at 4 °C. The resulting supernatant was then transferred to a new tube for subsequent use as the conditioned medium.

### Isolation and Identification, and adipogenic differentiation of FAPs

Animal experiments were performed under protocols approved by the Institutional Animal Care and Use Committee of local institution (AD2024060). All procedures adhered to the Guide for the Care and Use of Laboratory Animals. The isolation of FAPs followed protocols previously reported by other researchers (31). Briefly, intact hindlimb muscles were excised from C57/6J mice and transferred to pre-chilled PBS for brief preservation. After meticulous removal of non-muscle tissues, the sample was minced and digested with 0.2% type II collagenase (Beyotime, China) at 37 °C for 1h. The resulting lysate was filtered sequentially through 100-μm and 40-μm cell strainers (BD Bioscience, NJ, USA), followed by erythrocyte lysis buffer (Beyotime, China) at 4 °C for 5 minutes to remove red blood cells. Cells were resuspended in PBS containing 2% FBS, washed, and incubated with antibodies at 4 °C for 30 minutes prior to sorting. FAPs were identified as cells expressing CD31^-^CD45^-^ (Lin^-^)/α7INTEGRIN^-^/Sca-1^+^. To promote adipogenic differentiation, FAPs were cultured for up to three days in an induction medium composed of DMEM supplemented with 20% FBS, 0.5 mM IBMX (Sigma-Aldrich), 0.25 μM dexamethasone (Sigma-Aldrich), and 10 μg/ml insulin (Procell). Thereafter, the cells were maintained in a secondary medium containing DMEM with 10% FBS and 10 μg/ml insulin.

### Cell counting kit-8 assay

The CCK-8 assay was quantified using a CCK-8 kit (Beyotime, China). Cells were seeded into 96-well plates with a confluence of 80%. Cells were subsequently treated different interventions for 24 h. After treatment, the supernatant was removed, wells were rinsed with sterile PBS, and 100 µL of fresh medium containing 10 µL of CCK-8 reagent was added per well. After incubation for 2h, the absorbance at 450 nm was measured.

### Quantitative polymerase chain reaction

Total RNA was isolated from cells. RNA concentration was measured with a NanoDrop. Subsequently, RNA was reverse-transcribed into cDNA. Quantitative PCR amplification was performed on a ViiATM 7 Real-Time PCR System. The mRNA primer sequences were listed in **Supplementary Table 1**.

### scRNA-seq data processing & cell type identification

Single cell-sequencing (scRNA-seq) data of immobilized vs normal control skeletal muscle from mice lower limb was analyzed. Dataset was obtained via personal contact. The Seurat package (v5.3.0) in R (v4.4.0) was used for stringent quality control and filtering of scRNA-seq data. Exclusion criteria were: 1) the number of unique molecular identifiers (UMIS) detected in each cell (i.e., base factor) was lower than 300 or higher than 4500; 2) The proportion of mitochondrial gene expression exceeded 20%. All cell expression data after integration were standardized, and hypervariable genes were identified. Principal component analysis (PCA) was used for linear dimensionality reduction, and t-distributed stochastic neighbor embedding (t-SNE) and uniform manifold approximation and projection (UMAP) were performed. The identification of cell types was based on the differential expression of known cell type marker genes among clusters. After stringent quality control, a total of 67249 cells were retained, accounting for 95.7% of the original raw data. All cell expression data after integration were standardized using the LogNormalize method, and hypervariable genes were identified.

### Functional analyses and pathway activity scoring

Cellchat R package (v1.6.1) was employed to infer and compare the intercellular communication network. Kyoto Encyclopedia of Genes and Genomes (KEGG) and Gene Ontology (GO) functional enrichment analyses were performed using the clusterprofiler R package (v4.14.6). The stemness score of each subpopulation was calculated using the CytoTRACE algorithm (via the cytotrace R package). Pseudotemporal analysis was performed using the monocle3 R package (v1.3.7). Gene sets representing the core pathways of necroptosis, pyroptosis, and ferroptosis were curated from published literature. For each FAP, pathway activity scores were calculated using the AddModuleScore function in the Seurat package.

### Ferroptosis pathway regulation analysis design

To explore the potential regulatory mechanism of ferroptosis pathway in FAPs under immobilization, we performed the following specific analysis: first, The reported ferroptosis suppressor genes and driver genes were obtained respectively( http://www.zhounan.org/ferrdb/). Secondly, based on the single-cell RNA seq data, the differential expression analysis of FAPs cells in the IM and the NC was carried out.

Identification of Immobilization-Associated Ferroptosis Genes.

To identify ferroptosis-related genes whose expression changes were consistent with the immobilization phenotype, we performed two separate intersections using R: 1) between the ferroptosis suppressor gene set and the upregulated gene set, and 2) between the ferroptosis driver gene set and the downregulated gene set.

### PPI network construction & visualization

To construct protein-protein interaction (PPI) networks for the two sets of overlapping genes, the respective gene lists were submitted to the STRING database (version 12.0; https://string-db.org). The obtained interaction network data (TSV format) was then imported into Cytoscape software (version 3.7.1) for visualization and core node mining.

### Statistical analysis

All statistical analyses were performed using SPSS version 22.0 (IBM Corp., Armonk, NY, USA). Continuous variables were presented as mean ± standard deviation (SD), while categorical variables were expressed as counts or percentages. To explore the relationship between various clinical factors and the difference in quadriceps circumference at 3 months after surgery, t-test or paired t-test analyses were conducted as appropriate. Factors showing a significant correlation in univariate analysis were subsequently included in a multivariate linear regression model to identify independent predictors. A two-tailed P value < 0.05 was considered statistically significant.

## Results

26 patients including 13 males and 13 females were enrolled. The affected side was right in 18 cases (69.2%). The patients had a mean age of 30.46 ± 6.82 years, with a mean symptom duration of 1.86 ± 1.72 months. Graft types included autograft in 12 cases (46.2%) and artificial ligament in 14 cases (53.8%), with 17 (65.4%) undergoing concurrent meniscus repair. Pre-operative markers, including CRP and NLR, were also documented. The preoperative MFI was 6.42 ± 1.73%. 3 months after surgery, the mean decease in quadriceps circumference of the index knee was 1.82 ± 0.97 cm. In addition, 9 patients had MRI-based MFI value (5.08 ± 0.78%) of the contralateral knee, significantly lower than the index knee (7.66 ± 2.54%, p = 0.006). Detailed baseline characteristics were shown in **Table 1**.

**Table 1:** Basic characteristics of included patients.

Item	Value
Male/Female	13/13
Age (years)	30.46 ± 6.82
BMI (kg/m^2^)	24.82 ± 3.76
Left/Right	8/18
Duration (month)	1.86 ± 1.72
Autograft/Artificial ligament	12/14
C-reactive protein (mg/L)	2.58 ± 2.90
Neutrophil/Lymphocyte ratio	2.01 ± 0.65
Meniscus repair	17
Pre-operative MFI (%)	6.42 ± 1.73
Difference of quadriceps circumstance (cm)	1.82 ± 0.97

Next, the correlation between various factors and the quadriceps circumference decrease at 3 months after surgery were measured (**Table 2**). The results showed that preoperative CRP level (coefficient = 0.50, P = 0.008), concurrent meniscus repair (coefficient = 0.50, P = 0.009) and preoperative MFI (coefficient = 0.71, P < 0.001) were significantly correlated with the quadriceps circumference decrease. Therefore, these three factors were further included in the multivariate linear regression model (**Table 3**). The results showed that preoperative CRP level (coefficient = 0.39, P = 0.001), concurrent meniscus repair (coefficient = 0.23, P = 0.048) and preoperative MFI (β = 0.62, P < 0.001) were independent influencing factors of the quadriceps circumference decrease at 3 months after surgery. For each 1-point increase in preoperative MFI, the quadriceps circumference difference increased by an average of 0.62 cm; for each 1 mg/L increase in preoperative CRP, the circumference difference increased by an average of 0.39 cm; patients who underwent concurrent meniscus repair had an average increase of 0.23 cm in circumference decrease.

**Table 2:** Correlation of different factors and the difference of quadriceps circumstance at 3 months after surgery.

Item	Coefficient	P value
Male/Female	0.10	0.62
Age (years)	-0.12	0.56
BMI (kg/m^2^)	-0.11	0.96
Left/Right	0.073	0.72
Duration (month)	-0.071	0.72
Autograft/Artificial ligament	-0.32	0.11
C-reactive protein (mg/L)	0.50	0.008*
Neutrophil/Lymphocyte ratio	-0.07	0.73
Meniscus repair	0.50	0.009*
Pre-operative MFI (%)	0.71	<0.001*

*, statistical significance.

**Table 3:** Multivariate regression analysis of different factors and the difference of quadriceps circumstance at 3 months after surgery.

Item	Coefficient	P value
C-reactive protein (mg/L)	0.39	0.001*
Meniscus repair	0.23	0.048*
Pre-operative MFI (%)	0.62	<0.001*

*, statistical significance.

The aforementioned findings suggested that preoperative MFI was a risk factor of postoperative quadriceps atrophy. Given that the activation of FAPs is the key event driving MFI, we further detected that potential regulators activating FAPs. MuSCs, as a key player in skeletal muscle homeostasis, not only participates in myogenesis, but also secrets a series of factors or extracellular vesicles that can influence surrounding cells (32). Previous findings also suggested that MuSCs had a transient proliferation with in 6h after immobilization (33), and underwent apoptosis afterwards (34). Therefore, we analyzed the interaction between MuSCs and FAPs.

**Figure 1:**
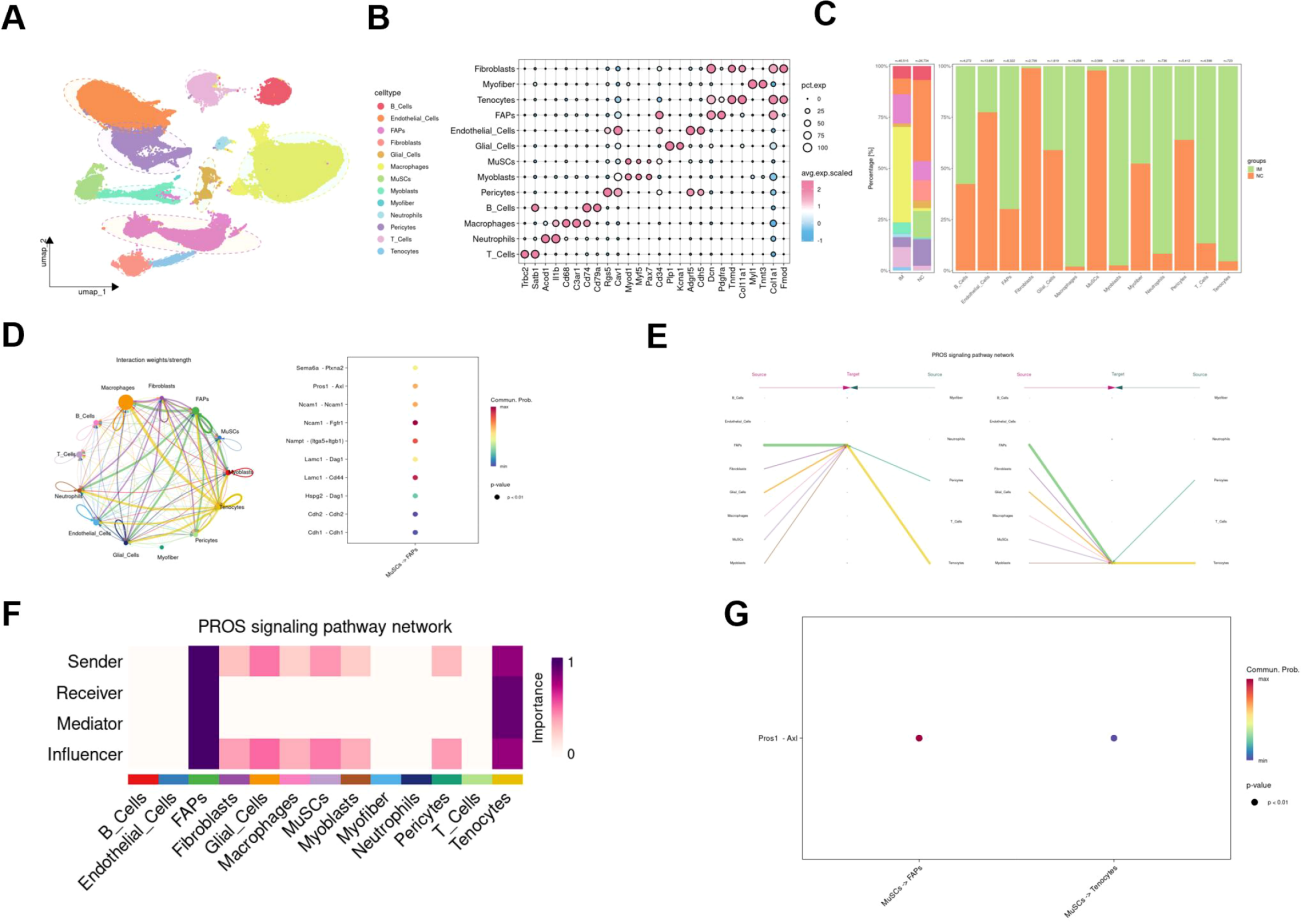
Characterization of the skeletal muscle microenvironment and MuSC–FAP interactions in skeletal muscle after immobilization. **(A)** UMAP visualizations of 67,249 single cells from skeletal muscle of immobilized and control mice after quality control. Cells are colored by annotated cell types. **(B)** Dot plot showing expression of canonical marker genes used for cell type identification. **(C)** Bar plot comparing the proportion of major cell types between IM and NC groups. **(D)** CellChat analysis revealing ligand–receptor interactions specifically from MuSCs to FAPs. Circle size represents communication probability. The size and color intensity of each bubble represent the communication probability (or interaction strength). **(E)** Contribution of each cell type to PROS signaling pathway activity inferred by CellChat. **(F)** Heatmap depicting the sending (sender) and receiving (receiver) roles of each cell type in PROS signaling. **(G)** MuSCs-specific signaling via the Pros1-Axl ligand-receptor pair. The size and color intensity of each bubble represent the communication probability (or interaction strength).

First, single-cell RNA sequencing comparing NC and IM group were re-analyzed. Dimensionality reduction and clustering analysis (t-SNE and UMAP) of the gene expression data from all cells identified the major cell types present in the skeletal muscle tissue (**Figure 1A**). FAPs (Pdgfra, Dcn), fibroblasts (Col1a1, Fmod), myoblasts (Myod1, Myf5), muscle stem cells (MuSCs; Pax7, Cd34), tenocytes (Tnmd, Col11a1), endothelial cells (Adgrf5, Cdh5), glial cells (Plp1, Kcna1), pericytes (Rgs5, Cav1), B cells (Cd74, Cd79a), macrophages (Cd68, C3ar1), neutrophils (Acod1, Il1b), and T cells (Trbc2, Satb1) were identified (**Figure 1B**). Analysis of the proportion of cell groups revealed that the proportion of MuSCs, FAPs and immune cells in the immobilization group increased, while the proportion of MuSCs decreased (**Figure 1C**).

In order to explore the potential regulatory role of MuSCs on FAPs, ligand-receptor interactions from MuSCs to FAPs were delineated using the CellChat database (**Figure 1D**). The analysis identified multiple significant signaling pathways from MuSCs to FAPs, many of which are known to have potent pro-survival functions (**Figure 1E**). Specifically, PROS pathway was known to be related to cell proliferation and differentiation (35). The main involved direction was from MuSCs to FAPs (**Figure 1F**), with Axl expression highly specific to FAPs (**Figure 1G**).

**Figure 2:**
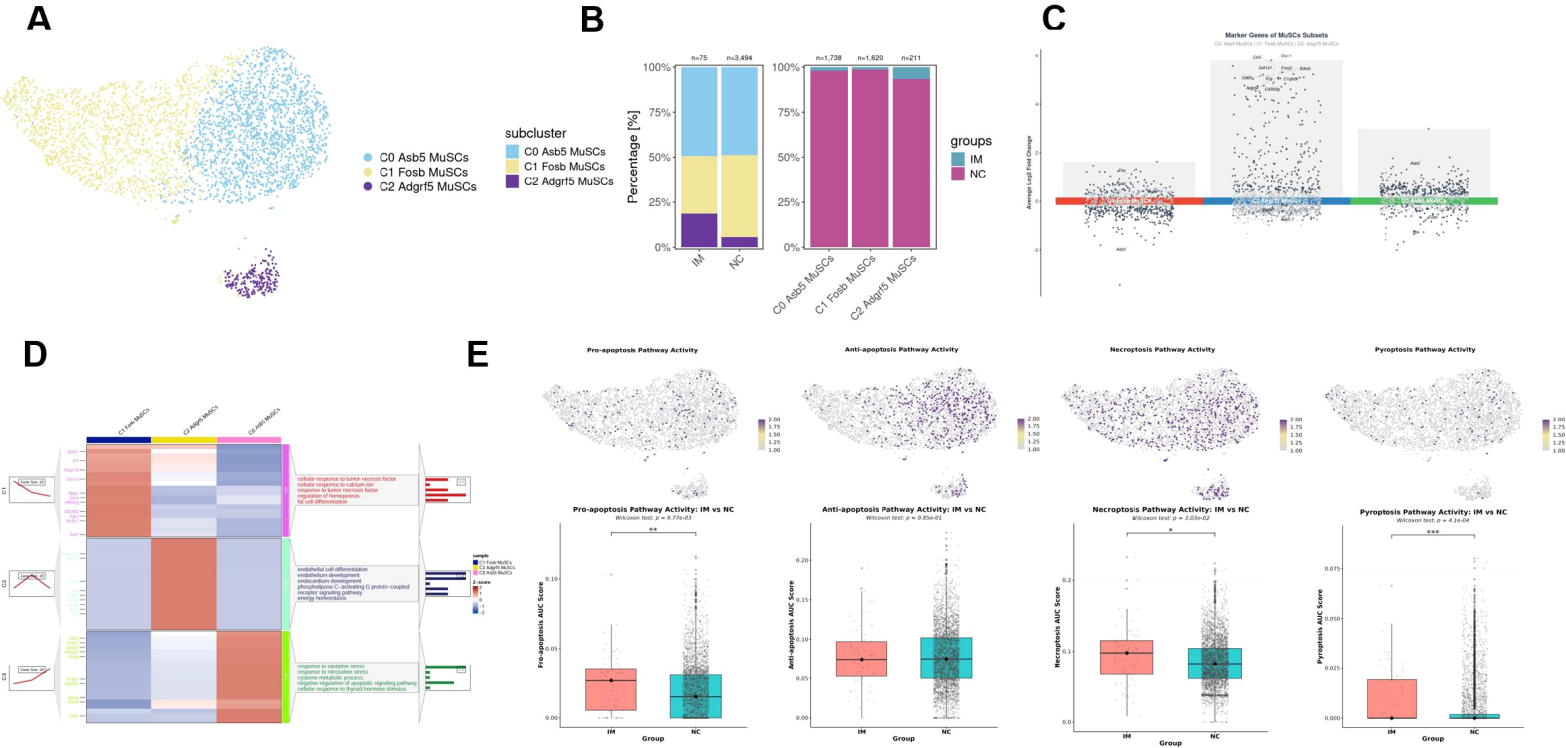
Subcluster characterization and functional dynamics of MuSCs following immobilization. **(A)** UMAP plot of MuSCs subclustering, identifying four distinct subpopulations (C0–C2) marked by high expression of Ccn4, Chodl, H19, and Myog, respectively. Cells are colored by subclusters. **(B)** Proportional distribution of MuSCs subclusters in IM and NC groups. **(C)** Volcano plots of highly variable genes in each MuSCs subcluster. **(D)** KEGG pathway enrichment analysis of highly variable genes in each MuSCs subcluster. **(E)** UMAP visualization of cell death pathway activities in MuSCs and comparison of cell death pathway activities between IM and NC groups.

Further subclustering analysis of MuSCs was carried out, and three subpopulations were successfully distinguished, with high expression of Asb5 (C0), Fosb (C1) and Adgrf5 (C2), respectively (**Figure 2A**). Among them, the number of satellite cells in the IM group is relatively low (**Figure 2B**).

Variable genes of each MuSC subpopulation were shown in **Figure 2C**. KEGG enrichment analysis of hypervariable genes in each subpopulations suggested different characteristics of four subpopulations (**Figure 2D**). The activity scores of pro-apoptotic genes, necroptotic genes, and pyroptotic genes in satellite cells of the IM group were all higher than those in the NC group (**Figure 2E**). There were no significant differences in the gene activity scores of the three programmed cell death modalities among the satellite cell subpopulations (**Supplementary Figure 1A**).

**Figure 3:**
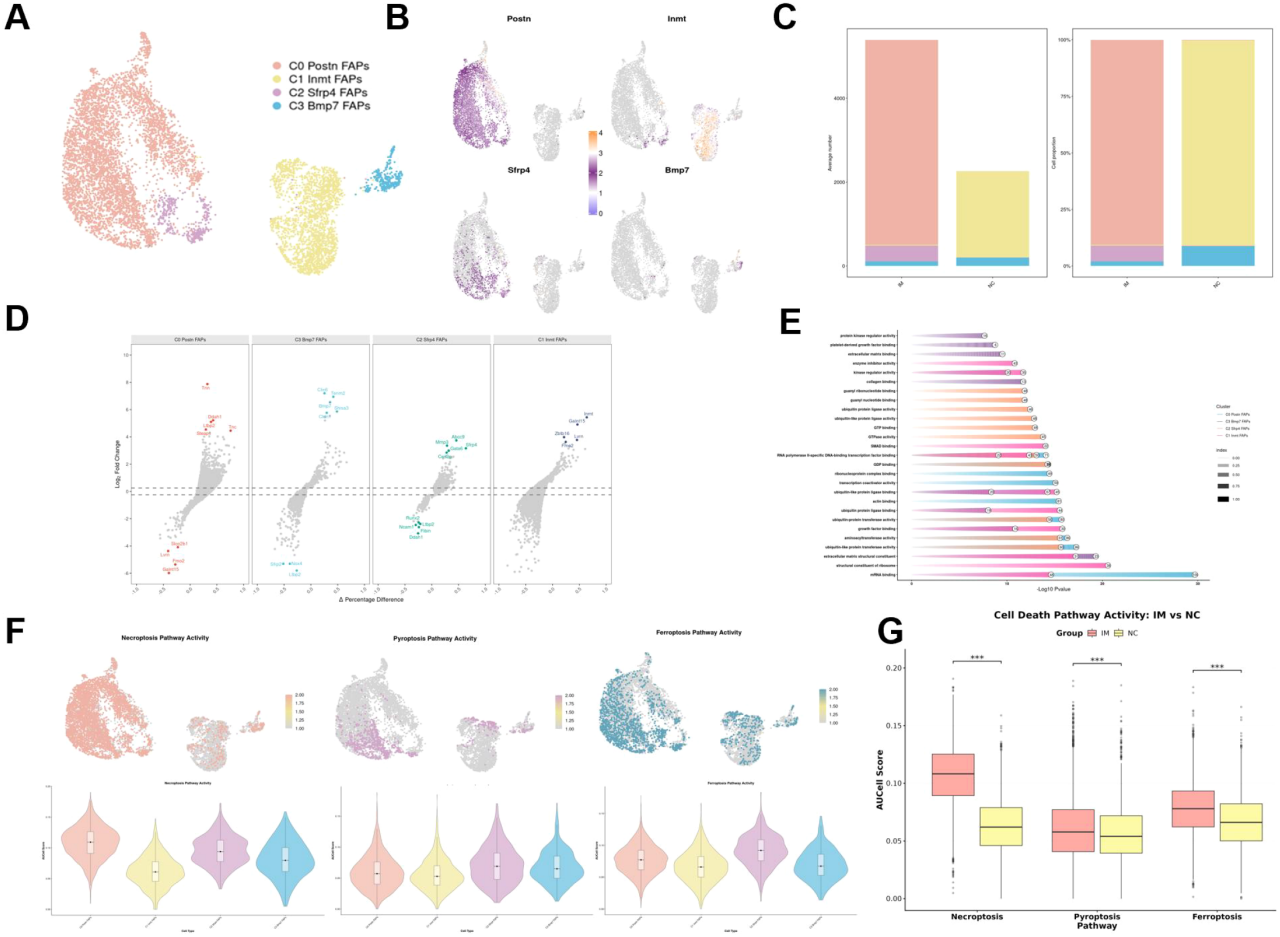
Subcluster characterization and functional dynamics of FAPs following immobilization. **(A)** UMAP plot of FAPs subclustering, defining four subpopulations. **(B)** Feature plots showing expression of representative marker genes (Postn, Inmt, Sfrp2, Bmp7) for each FAPs subcluster. **(C)** Stacked bar chart showing the proportion of FAP subclusters in IM and NC groups. **(D)** Volcano plots highlighting differentially expressed genes in each FAP subcluster. **(E)** GO enrichment analysis of highly variable genes in each FAP subcluster. **(F)** Activity scores of three programmed cell death pathways (necroptosis, pyroptosis, and ferroptosis) across FAP subclusters. **(G)** Comparison of cell death pathway activities between IM and NC groups. ***: p < 0.001.

Four subpopulations of FAPs were also identified (**Figure 3A**), with related marker genes (**Figure 3B**). The IM group was mainly composed of C0, while the NC group was mainly composed of C1 (**Figure 3C**). Hypervariable genes of each subpopulation were shown in **Figure 3D**. Ontology (GO) enrichment analysis of hypervariable genes in each subpopulation uncovered distinct functional profiles (**Figure 3E**).

Given that FAPs were aberrantly activated in MFI, we systematically evaluated the activity distribution of three programmed cell death pathways (necroptosis, pyroptosis, and ferroptosis) in FAPs (**Figure 3F**). The activity scores for all three pathways were significantly enriched in the IM group, indicating potential involvement (**Figure 3G**).

**Figure 4:**
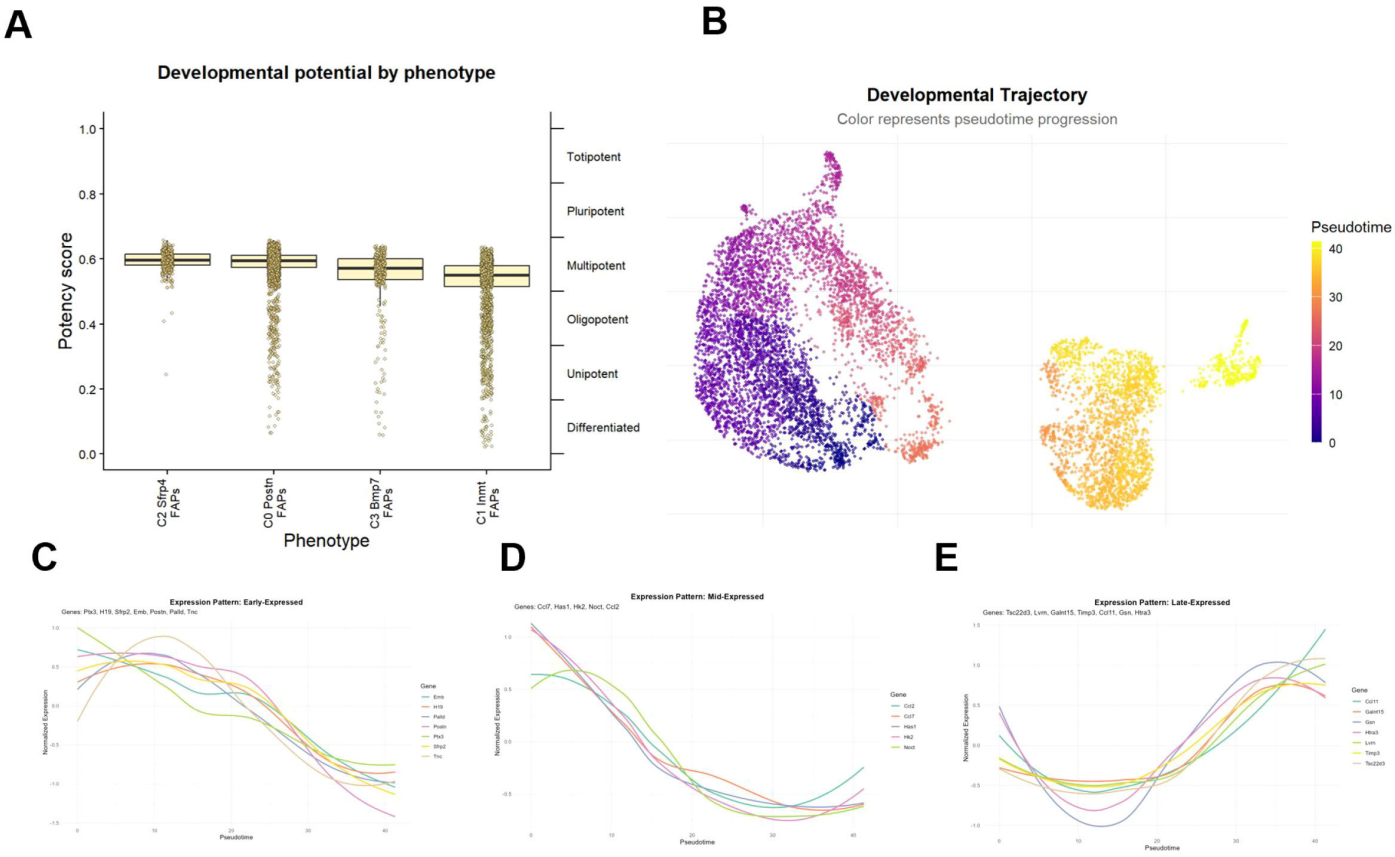
Pseudotemporal trajectory analysis of FAPs. **(A)** CytoTRACE stemness scores across FAP subclusters. **(B)** Pseudotime trajectory of FAPs. **(C–E)** Heatmap of gene expression modules along pseudotime. Early phase (pseudotime 0–15), Middle phase (15–25), Late phase (25–40).

To describe the functional state transition of FAPs under immobilization, we first calculated the stemness score by CytoTRACE, and the results indicated that C0 and C2 subpopulations had the highest stemness (**Figure 4A**). Subsequently, pseudotemporal analysis was performed, and the two subpopulations of the IM group were distributed in the early stage of the trajectory, while the NC group was distributed in the late stage of the trajectory (**Figure 4B**). In addition, we identified three types of gene modules with clear temporal expression characteristics. At the early stage of the trajectory (pseudo time 0-15), a group of genes are specifically activated, including extracellular matrix remodeling related genes (Postn, Tnc, Palld) and development and morphogenesis related genes (Emb, H19) (**Figure 4C**). In the middle of the trajectory (pseudo time 15-25), transient high expression of chemokine genes (Ccl2, Ccl7) was noticed (**Figure 4D**). In the late stage (pseudo time 25-40), the expression of early activated genes decreased, while a group of novel functional genes continued to be upregulated, including matrix protease inhibitors (Timp3, Gsn, Htra3) and immune regulatory factors (Tsc22d3, Ccl11) (**Figure 4E**).

**Figure 5:**
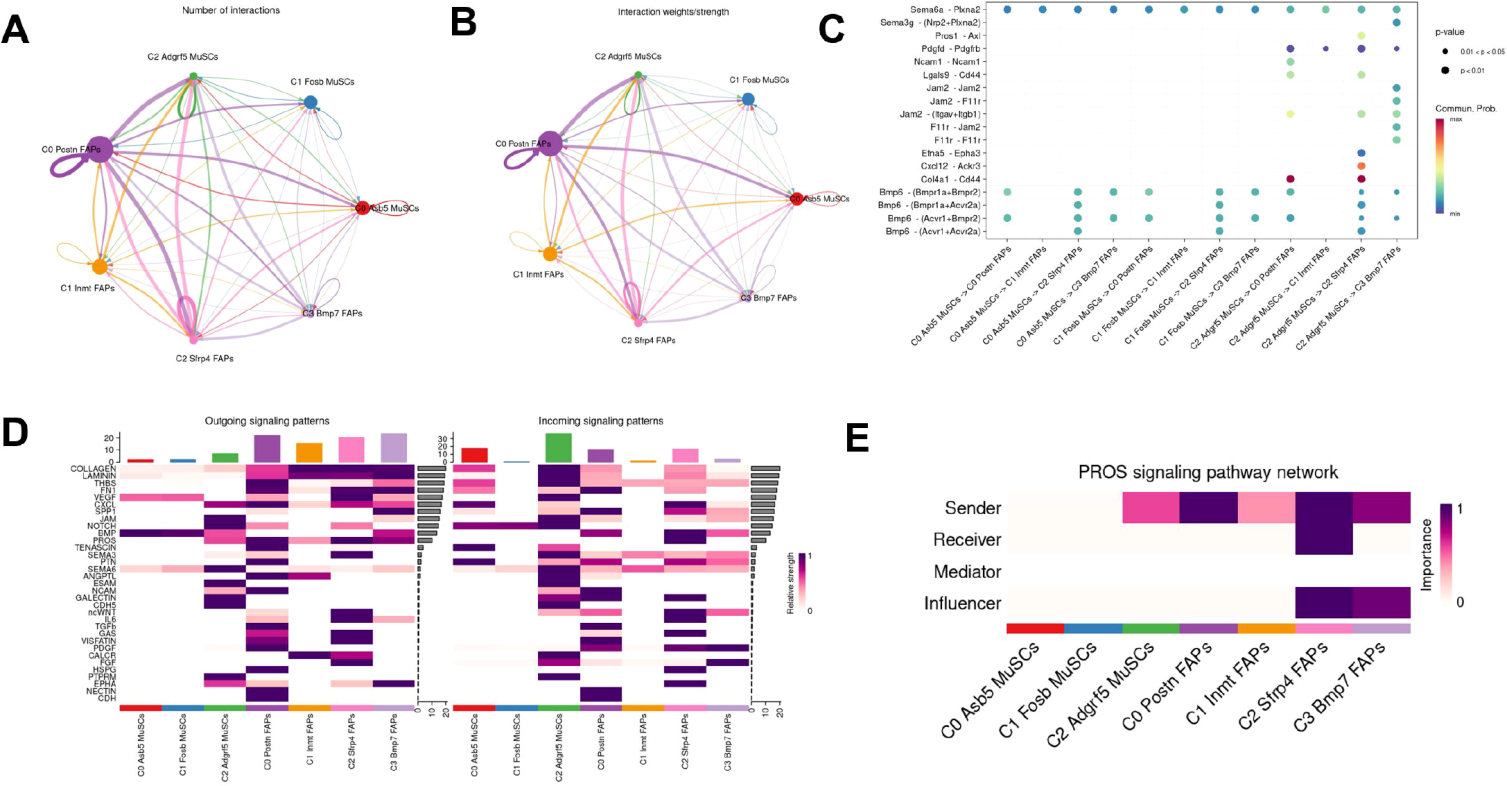
CellChat analysis performed on the subdivided MuSCs and FAP subclusters to infer potential intercellular communication. **(A, B)** CellChat analysis of interactions between MuSCs and FAP subclusters. **(C)** Bubble plot visualizing ligand-receptor interactions between the subdivided MuSCs subclusters and FAP subclusters. The size and color intensity of each bubble represent the communication probability (or interaction strength). **(D)** Outgoing and incoming interaction strengths for each subcluster. **(E)** Roles of subclusters in PROS signaling: MuSCs C2 and FAPs are primary senders; FAP C2 is primary receivers; FAP C2 and C3 act as key influencer and mediator.

To further delineate the intercellular communication between MuSC and FAP subpopulations, we performed Cellchat analysis on the re-clustered datasets (**Figures 5A–D**). In the PROS pathway, C2 subpopulations of muscle satellite cells and FAPs were the key initiator, while C2 subpopulations of FAPs were the main receivers, and the C2 and C3 subpopulation was the main influencer (**Figure 5E**).

**Figure 6:**
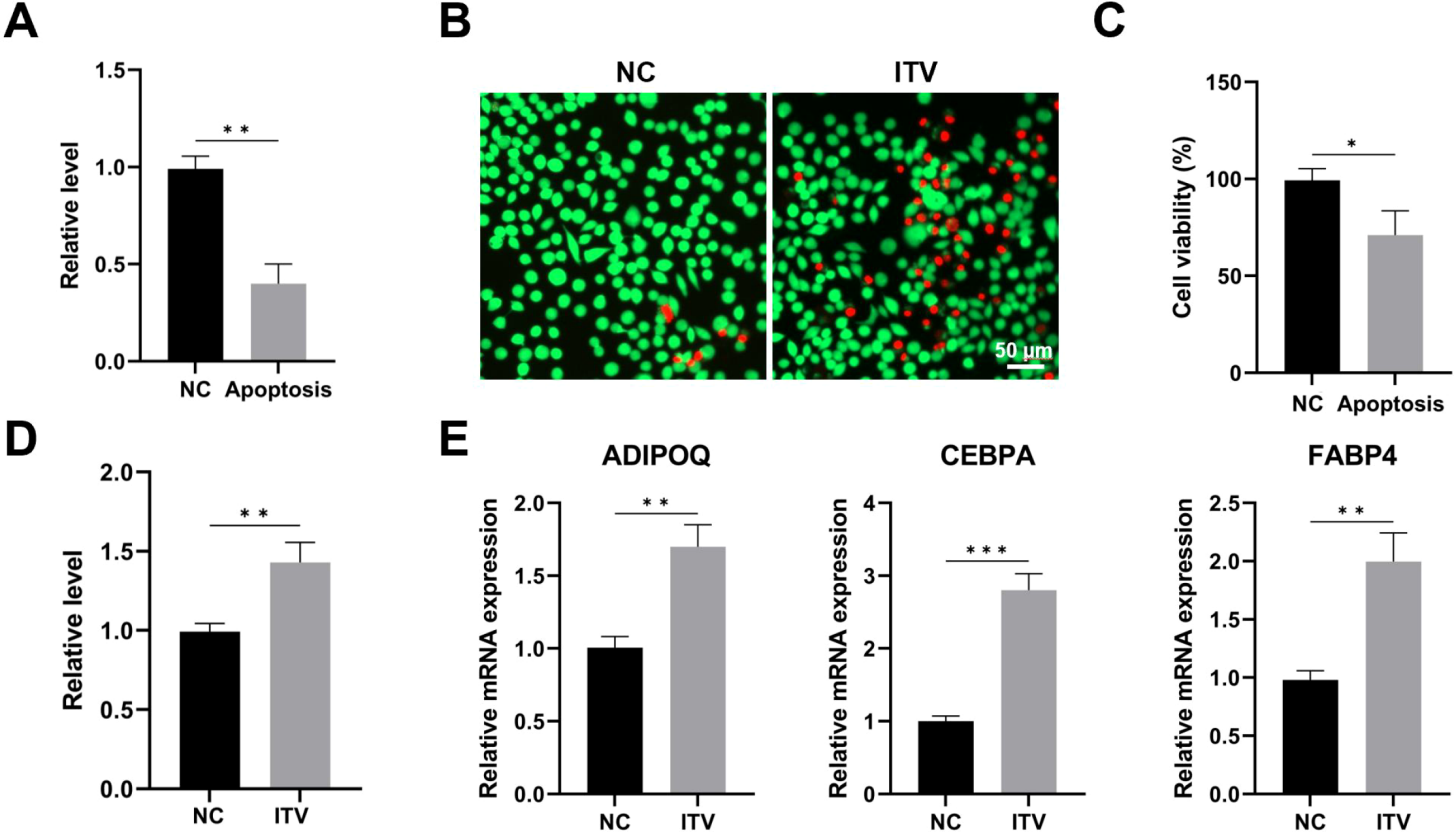
Condition medium of apoptotic muscle satellite cells promotes FAPs activation and adipogenesis. **(A)** CCK-8 assay of muscle satellite cells with or without induction of apoptosis. **(B, C)** Live (green) and dead (red) image of muscle satellite cells with or without induction of apoptosis, and related quantification. **(D)** CCK-8 assay of FAPs with or without stimulation of condition medium of apoptotic muscle satellite cells. **(E)** PCR of the level of adipogenic markers of FAPs with and without stimulation of condition medium of apoptotic muscle satellite cells. *: p < 0.05; **: p < 0.01; ***: p < 0.001. ITV: Intervention.

Based on these findings in silico, we conducted a series of verifications (**Figure 6A**). Apoptosis of MuSCs cells were first induced to mimic the apoptotic status of MuSCs in immobilized skeletal muscle (**Figures 6B, C**). Then the condition medium was harvested to educate FAPs. Interestingly, both the cell viability (**Figure 6D**) and adipogenic markers (Adipoq, Cebpa, Fabp4) (**Figures 6E**) were enhanced under this stimulation, suggesting that the secretome of apoptotic MuSCs may positively regulate FAPs activity, thus inducing MFI.

**Figure 7:**
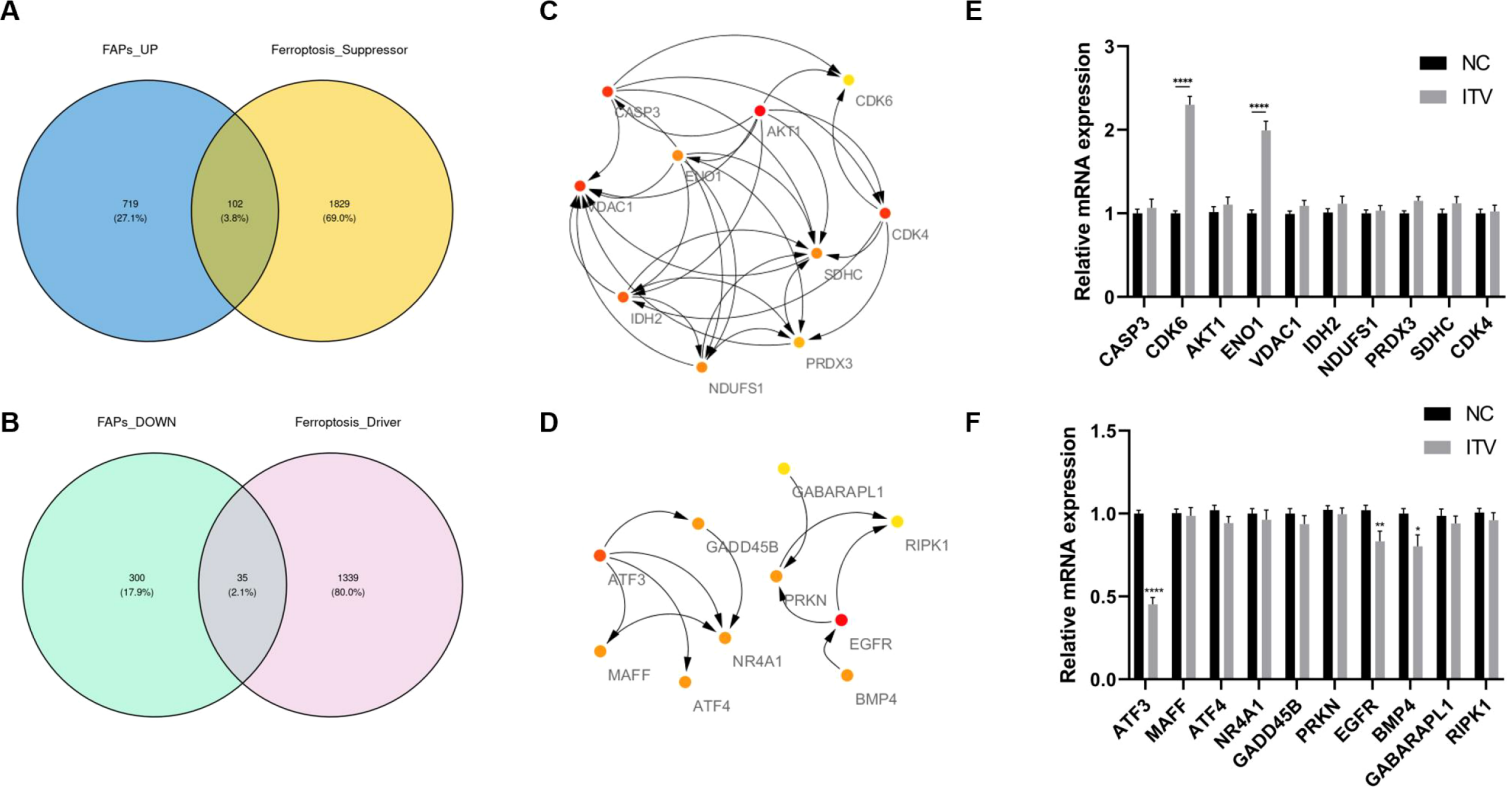
Identification of ferroptosis-related core genes in FAPs after immobilization. **(A)** Overlap between ferroptosis suppressor genes and up-regulated DEGs in the IM group (102 overlapping genes). **(B)** Overlap between ferroptosis driver genes and down-regulated DEGs in the IM group (35 overlapping genes). **(C)** The top 10 hub genes from the suppressor-upregulated network, ranked by connectivity. **(D)** The top 10 hub genes from the driver-downregulated network, ranked by connectivity. **(E)** Comparisons of the hub suppressor genes expression between normal and activated FAPs. **(F)** Comparisons of the hub driver genes expression between normal and activated. **(F)** Comparisons of the hub driver genes expression between normal and activated FAPs. *, p < 0.05; **, p < 0.01; ****, p < 0.0001; ITV, Intervention; *, p < 0.05; **, p < 0.01; ****, p < 0.0001; ITV, Intervention.

As a major type of cell death, ferroptosis is significantly inhibited in FAPs in immobilized muscle (36). Therefore, we intersected the suppressor and driver genes of the ferroptosis pathway with the DEGs in FAPs between the IM and NC group. Since ferroptosis was inhibited during immobilization, we mainly focused on up-regulated suppressors and down-regulated drivers. The intersection of suppressor and the up-regulated genes in the IM group yielded 102 genes (**Figure 7A**), and the intersection of driver and the down regulated genes yielded 35 genes (**Figure 7B**). Subsequently, protein-protein interaction (PPI) networks for these overlapping gene sets were constructed using the STRING database (**Supplementary Figures 2A**, **S2B**). Hub genes based on network centrality metrics were generated (**Figures 7C, D**), and the expression difference between FAPs and activated FAPs were detected. Several drivers and suppressors were significantly abnormally expressed (**Figures 7E, F**), indicating potential involvement of these genes in FAPs activation under immobilized status.

## Discussion

Previous studies have extensively documented fat infiltration in the hamstring muscles following ACLR. For instance, one study of patients who underwent ACLR demonstrated that the volume and cross-sectional area of the semitendinosus and gracilis muscles continued to decrease 9 to 11 years postoperatively (21). Building upon this foundation, the present study further reveals that the quadriceps femoris muscle also exhibits fat infiltration following ACLR surgery, and this infiltration is independently associated with muscle strength.

In the observations of this study, fat infiltration in the quadriceps femoris exhibited a progressive characteristic. It was correlated with systemic inflammatory markers, CRP and NLR. As an acute-phase reactant, elevated CRP levels usually indicates the presence of inflammation (37). NLR is calculated by ratio of neutrophils to lymphocytes in the blood, reflecting systemic inflammation and immune status. An elevated NLR is commonly associated with more severe inflammatory states and poor prognosis (38). A study of individuals aged 50 and older found that CRP levels were negatively correlated with muscle strength in men (39). A cross-sectional study of hemodialysis patients in China also confirmed a significant association between NLR and the risk of sarcopenia (40). Another preoperative study on colorectal cancer patients found that sarcopenia and intramuscular lipid infiltration were significantly associated with elevated NLR (41). Our findings are consistent with the widely accepted conclusion from previous studies that inflammatory responses are closely associated with the pathophysiological processes of muscle atrophy and fat infiltration (42).

Notably, both preoperative MFI and CRP levels emerged as independent predictors of quadriceps atrophy in our multivariate model. While our study was not specifically designed or powered to assess interactive effects, it is clinically plausible that patients with concurrently elevated MFI and systemic inflammation may face a particularly high risk of postoperative muscle wasting—given that MFI likely reflects local chronic muscle degeneration, whereas CRP captures modifiable systemic inflammatory status. Future studies with larger sample sizes and prospective designs are warranted to formally test whether these two risk factors exert additive or synergistic effects, and whether their combination could improve risk stratification and guide personalized rehabilitation strategies. In addition, we unexpectedly found no significant association between preoperative NLR and 3-month postoperative quadriceps atrophy after ACLR, contradicting previous studies. This discrepancy may result from different timing of NLR measurement, distinct inflammatory properties between NLR and CRP (with CRP more stable), and limited statistical power from our sample size. Therefore, CRP may be a more reliable inflammatory biomarker than NLR for predicting postoperative muscle outcomes in subacute ACL reconstruction patients.

The positive correlation between MFI progression and quadriceps atrophy observed in our preliminary analysis suggests a mechanistic link: elevated preoperative MFI may reflect an activated adipogenic milieu within the muscle that persists or even amplifies after surgical insult. This finding underscores the clinical importance of assessing preoperative MFI as a potential therapeutic target. If confirmed in larger longitudinal studies, interventions aimed at mitigating preoperative MFI—such as early quadriceps strengthening or pharmacological modulation of adipogenic signaling—could help preserve postoperative muscle mass and improve functional recovery.

The cause of muscle fat infiltration is the abnormal activation of FAPs and adipocyte differentiation (43). This abnormal differentiation is common in muscle injuries and diseases, adversely affecting muscle function and the prognosis of various conditions (44). MME+FAPs are considered the main adipogenic cell population in human skeletal muscle fat infiltration, characterized by low extracellular matrix remodeling capacity and high adipogenic potential (45). On the other hand, FAPs also play a crucial role in muscle regeneration. They can secret many kinds of factors that support the proliferation and differentiation of muscle stem cells (MuSCs). Experiments by YU et al. found that muscles lacking FAPs exhibited reduced fat infiltration but also impaired regenerative capacity. This result indicates that FAPs play a crucial role in the repair process (46).

This study found that although elevated CRP levels are correlated with the progression of fat infiltration and inflammation, *in vitro* experiments demonstrated that CRP cannot directly stimulate the abnormal activation of FAPs. This suggested that the association between inflammation and abnormal FAP activation may be mediated by other indirect mechanisms or upstream factors. Previous studies have confirmed that ACLR surgery induces myocyte apoptosis (47). This means that CRP can directly stimulate myocyte apoptosis and indirectly influence muscle repair and fat infiltration by affecting myocyte survival.

Further analysis found that the conditioned medium from apoptotic MuSCs stimulates abnormal activation of FAPs, suggesting that MuSCs apoptosis may release certain signaling molecules, which in turn promote the adipogenic differentiation of FAPs. Our findings raise the key question of which factors in the apoptotic muscle satellite cells secretome promote FAP activation and adipogenic differentiation. Although full secretome characterization is beyond this study, CellChat analysis identifies PROS signaling as the primary candidate, with strong ligand–receptor pairing between apoptotic muscle satellite cells and FAPs. PROS acts paracrinely to stimulate FAP proliferation, enhance survival via PI3K/AKT and ferroptosis-related pathways, and prime adipogenic differentiation. Future unbiased proteomic studies will help dissect this complex signaling network. Previous studies have indicated that suppression of ferroptosis in FAPs is a key factor contributing to their abnormal activation (36). Based on the background, this study examined ferroptosis markers and found a significant decrease in their levels, confirming the inhibition of ferroptosis in FAPs. Our results further suggest that conditioned medium from apoptotic MuSCs may promote the abnormal FAP activation by suppressing ferroptosis in these cells. In our immobilized FAPs, downregulation of pro-ferroptotic drivers reduces lipid peroxidation triggers, while upregulation of anti-ferroptotic defenders enhances oxidative stress defense. This dual shift elevates the threshold for ferroptosis induction, allowing FAPs to resist cell death and persist in the immobilization-induced microenvironment. These surviving FAPs retain adipogenic potential and undergo differentiation in response to signals from apoptotic MuSCs. Thus, ferroptosis suppression acts as a permissive gatekeeper—enabling FAP survival long enough for adipogenic programs to be executed. Clinically, this suggests that modulating ferroptosis could alter the trajectory of muscle fatty infiltration, and this gene signature may serve as a biomarker for identifying patients at risk for poor muscle recovery after ACLR.

Apoptosis, as a form of programmed cell death, is important for maintaining organismal growth and tissue homeostasis (48–50). During apoptosis, a series of bioactive substances are released, including cytokines, chemokines, growth factors, and apoptotic bodies (51, 52). These secreted molecules may act as “messengers”, transmitting signals between cells and thereby influencing the behavior of surrounding cells (53).

Apoptotic bodies are membrane-bound extracellular vesicles formed during the disintegration of apoptotic cells, containing nuclear fragments, organelles, and cytoplasmic components. They participate in intercellular communication by transporting nucleic acids, proteins, lipids, and other biomolecules to target cells, thereby influencing cellular functions and behaviors (54). For example, apoptotic bodies can induce macrophage metabolic reprogramming through the PDL1-PD1 axis (55). Different subtypes of apoptotic vesicles derived from bone marrow mesenchymal stem cells (BMSCs) exhibit distinct functional diversity in tissue regeneration (56). Apoptotic bodies derived from mesenchymal stem cells (MSCs) have been shown to have therapeutic potential in immunomodulation and tissue regeneration (57).

Apoptotic bodies are rich in biomolecules such as miRNAs, proteins, and lipids. These molecules may undergo changes under disease conditions, making them potential diagnostic markers and therapeutic targets (48, 58, 59). Future research may focus on elucidating the differences in secretory components among different apoptotic pathways and their specific biological functions. It can systematically characterize the secretome of apoptotic MuSCs to identify specific mediators responsible for abnormal adipogenic differentiation.

Identifying apoptotic MuSC-derived factors as key mediators of FAP activation offers translational potential for ACL injury, aligning with evidence that satellite cell depletion post-ACLR disrupts muscle regenerative capacity and promotes fibroadipogenic infiltration (60, 61). Apoptotic vesicles in circulation or synovial fluid may serve as non-invasive biomarkers for muscle degeneration, while potential therapeutic strategies include inhibiting apoptotic body formation, blocking their uptake by FAPs, or targeting downstream PROS signaling—approaches that, despite requiring preclinical validation, provide a framework for translating mechanistic findings into clinical practice. Considering the tremendous potential of apoptotic bodies in disease diagnosis and treatment, developing highly specific detection techniques is crucial for early diagnosis and monitoring disease progression (62). Leveraging apoptotic bodies as natural carriers enables the design of targeted therapeutic strategies. For instance, apoptotic bodies can be employed in the treatment of multiple myeloma by restoring Fas-mediated apoptosis to inhibit tumor growth (63). Engineered apoptotic bodies also offer novel tools for drug delivery and immunotherapy (64). Through gene editing technologies such as the CRISPR/Cas system, cells can undergo precise genetic modification, thereby influencing the biological activity and contents of apoptotic bodies (65). This opens possibilities for developing more specific and efficient therapeutic approaches. In the future, this approach may also become a key research focus for exploring adjunctive therapies to improve muscle-fat infiltration and muscle atrophy observed following ACLR surgery.

Several limitations of this study should be acknowledged. First, the relatively small sample size (n = 26) limits the generalizability of the current findings, and we will therefore initiate a multicenter expansion to validate the observed correlations. Second, all functional and mechanistic experiments were performed using mouse-derived cell lines (MuSCs and primary mouse FAPs). Although this approach allowed us to establish a preliminary mechanistic framework, the direct extrapolation of these findings to human biology remains to be verified. Future validation using human-derived cells—such as FAPs and MuSCs isolated from discarded hamstring tendons during ACL reconstruction surgery—will be essential to confirm the translational relevance of the proposed mechanisms. Third, the study relied solely on single-cell RNA sequencing to investigate muscle immobilization, and complementary omics approaches such as proteomics or metabolomics were not incorporated. A more comprehensive multi-omic perspective, particularly regarding FAPs stimulated with apoptotic muscle-satellite-cell-conditioned medium, would provide deeper insight into the underlying biological processes and should be considered in future research. Fourth, while we identified a set of differentially expressed genes under immobilization-mimicking conditions, the present study does not functionally dissect which of these genes are the key drivers of the observed phenotypic changes. Additional loss- and gain-of-function studies are required to pinpoint the critical molecular mediators and to establish causality. Fifth, detailed meniscal tear type and repair technique data were not available for all patients due to the retrospective design and variability in surgical record documentation. While all meniscal repairs were performed using the Fast-Fix system (Smith & Nephew) and all patients followed a standardized postoperative rehabilitation protocol, we cannot entirely rule out the possibility that subtle differences in meniscal pathology or repair technique may have influenced quadriceps recovery. Sixth, this study did not include patient-reported functional outcome scores (e.g., Lysholm score or IKDC subjective form). The 3-month follow-up period represents an early postoperative phase when patients are typically still progressing toward full squatting and functional recovery, and functional scores at this stage may be influenced by transient factors such as pain, effusion, or rehabilitation status, and may not reliably reflect the long-term impact of muscle changes. Seventh, based on current findings, several ferroptosis-related genes were identified as potential regulators in apoptotic-muscle-satellite-cell-induced FAPs activation. Further evidences are needed to locate the exact key gene involved in this process.

In conclusion, our research found that, pre-operative MFI and inflammation level, as indicated as C-reactive protein, are independent risk factors for muscle atrophy following ACLR. Abnormal activation of FAPs with enhanced adipogenesis ability is stimulated by the secretome of MuSCs, which were apoptotic during immobilization.

## Data Availability

The data analyzed in this study is subject to the following licenses/restrictions: Dataset of single cell sequencing was retrieved via personal contact from a previously published paper【PMID: 40316864. Requests to access these datasets should be directed to Jiale Tan, tanjiale2873@163.com.
