# Supplementary material for: Preoperative systemic inflammation and muscle fatty infiltration are prognostic factors for quadriceps atrophy following anterior cruciate ligament reconstruction
Source: Front Immunol. 2026 Mar 25;17:1796054. doi: 10.3389/fimmu.2026.1796054 (PMC13056832; doi:10.3389/fimmu.2026.1796054)
Supplement: Supplementary file 1 [file DataSheet1.docx]

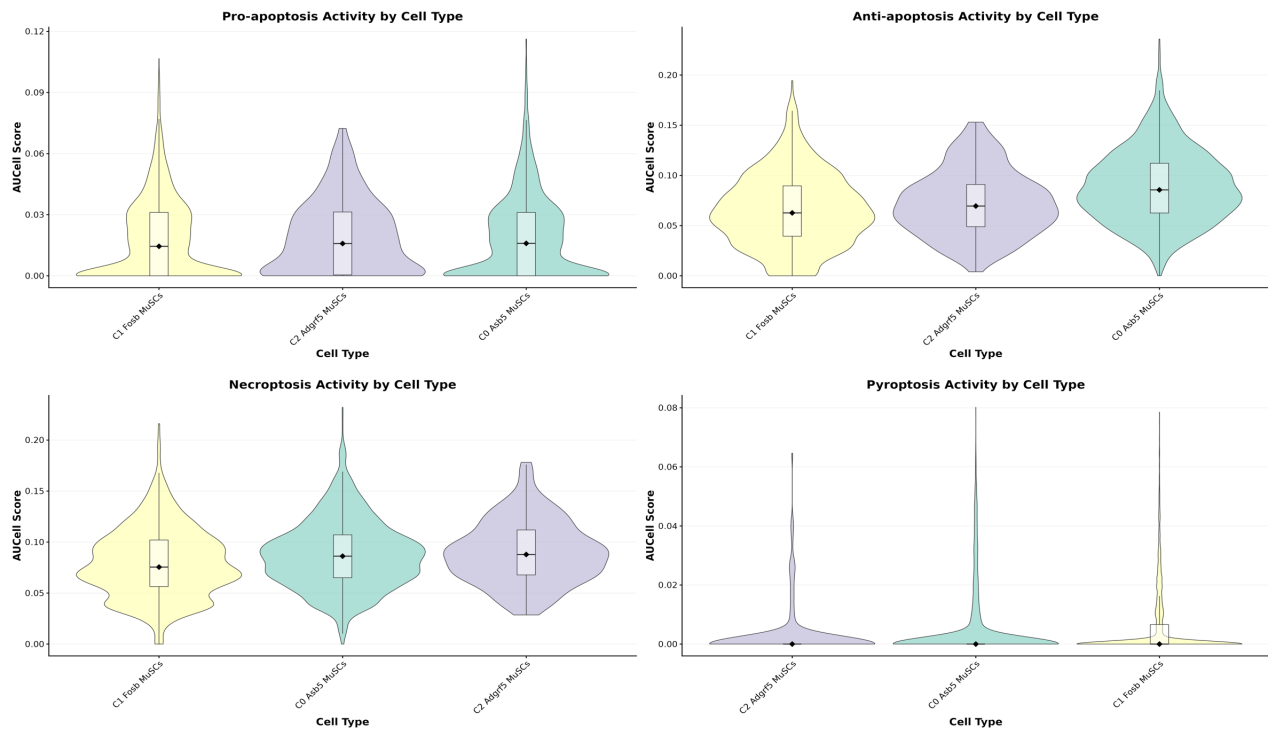


Figure S1 Activity scores of three programmed cell death pathways across FAP subclusters.


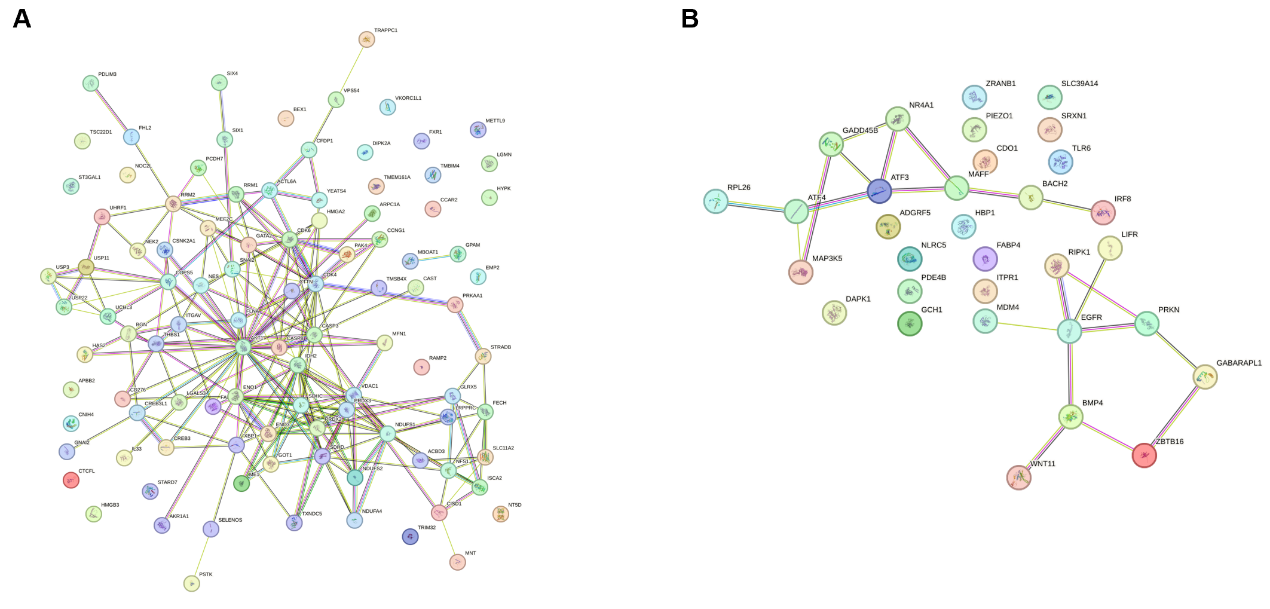


Figure S2 PPI network of the genes at the intersection of ferroptosis-related genes and differentially expressed genes (DEGs) in FAPs.

(A) PPI network of the 102 genes overlapping with ferroptosis suppressors.

(B) PPI network of the 35 genes overlapping with ferroptosis drivers.

Table S1 Primers of genes.

| Gene Name | Sequences |  |
| --- | --- | --- |
| CDK4 | Forward | 5'-ATGGGACGGTGTACAAAGCCCG-3‘ |
|  | Reverse | 3'-CCAAAGTCAGCCAGCTI'GACGG-3’ |
| CDK6 | Forward | 5‘-GACCTACTTCTGAAATGCCTGACG-3’ |
|  | Reverse | 5‘-GAGGTGCTCTGGTTGGATGG-3’ |
| SDHC | Forward | 5ʹ- CTGTTGCTGAGACACGTTGGT-3ʹ |
|  | Reverse | 5ʹ-ACAGAGGACGGTTTGAACCTA-3ʹ |
| CASP3 | Forward | 5‘-AGCTTGGAACGGTACGCTAA-3’ |
|  | Reverse | 5‘-CGTCCACATCCGTACCAGAG-3’ |
| VDAC1 | Forward | 5‘-GGGATGTCTTCACCAAGGGCTAC-3’ |
|  | Reverse | 5‘-TGGTCTCCGTGTTGGCAGAAC-3’ |
| AKT1 | Forward | 5‘-TCCAGGGCCAAAGTCCAGCAAGAA-3’ |
|  | Reverse | 5‘-CCAGAGGGAGAGGGCCAGTTAGCA-3’ |
| ENO1 | Forward | 5′-CGGCTTTACGTTCACCTCGG-3′ |
|  | Reverse | 5′-TCAACAGCCTTTGAGACACCCT-3′ |
| IDH2 | Forward | 5’-AGTGTGGCTGTCAAGTGTG-3’ |
|  | Reverse | 5’-AAGGATGTTCCGGATCGTTC-3’ |
| PRDX3 | Forward | 5′-GAGATGCTCTGTGGTCACGC-3′ |
|  | Reverse | 5′-GTCTGCCAGTTTACAGTGACCC-3′ |
| NDUFS1 | Forward | 5‘-TCCAGTGTACCCGGTGCATC-3’ |
|  | Reverse | 5‘-AATGTATGTGCCAACTTGCATGTC-3’ |
| EGFR | Forward | 5'-CTTCCAGGAGGAGGAGAGAAA − 3' |
|  | Reverse | 5'-CTGGGAGGAGGAGGAGAG − 3' |
| BMP4 | Forward | 5‘-GCCGGGGCCATACCTTGAC-3’ |
|  | Reverse | 5‘-ATGGCGACGGCAGTTCTTATTCTT-3’ |
| PRKN | Forward | 5‘-GTCTTCCAATGTAACCACCGC-3’ |
|  | Reverse | 5‘-GGAGTAGCCAAGTTGAGCGTC-3’ |
| GABARAPL1 | Forward | 5‘-GAGGACCACCCCTTCGAATATC-3’ |
|  | Reverse | 5‘-CAGTGAGGTCGGAGGGCA-3’ |
| ATF3 | Forward | 5‘-ATTCGCCATCCAGAACAAGC-3’ |
|  | Reverse | 5‘-CCACCTCAGACTTGGTGACT-3’ |
| GADD45B | Forward | 5‘-TGCTCTTGGGGATCTTCCGT-3’ |
|  | Reverse | 5‘-CTGCATCTTCTGAACCGCGT-3’ |
| NR4A1 | Forward | 5′-CACCCACTCTCCACCT-3′ |
|  | Reverse | 5′-TCCCCACACACACCACA-3′ |
| ATF4 | Forward | 5‘-CTGAACAGCGAAGTGTTGGC-3’ |
|  | Reverse | 5‘-AAAAGGCATCCTCCTTGCCG-3’ |
| MAFF | Forward | 5‘-GTGGATCCCTTATCCAGCAAAG-3’ |
|  | Reverse | 5‘-CATCAGCGCTTCATCCGA-3’ |
| GABARAPL1 | Forward | 5‘-CCAGTTGTGGCAGGAGACAT-3’ |
|  | Reverse | 5‘-GCAATCATAACCGTTCCCGC-3’ |
| Adiponectin (AdipoQ) | Forward | 5‘-AGATGGCACTCCTGGAGAGAAG-3’ |
|  | Reverse | 5‘-ACATAAGCGGCTTCTCCAGGCT-3’ |
| FABP4 | Forward | 5’-GGATGGAAAGTCGACCACAA-3’ |
|  | Reverse | 5’-TGGAAGTCACGCCTTTCATA-3’ |
| CEBPA | Forward | 5′-GCGGGAACGCAACAACATC-3′ |
|  | Reverse | 5′-GTCACTGGTCAACTCCAGCAC-3′ |
